# Effects of alfalfa fermented feed on growth performance, meat quality, and fecal microflora of Taoyuan Black pigs

**DOI:** 10.3389/fvets.2025.1689943

**Published:** 2025-12-01

**Authors:** Yongfei Zhu, Xinyu Lei, Xiaoya Zhang, Yongmei Wu, Biguang Lv, Xuan Cheng, Haoqian Cheng, Li Wang, Yinghui Li, Cong Li, Zhifei Zhang, Qian Lin

**Affiliations:** 1 College of Animal Science and Technology, Hunan Agricultural University, Changsha, China; 2 Institute of Bast Fiber Crops, Chinese Academy of Agricultural Sciences, Changsha, China; 3 Hunan Provincial Key Laboratory of the Traditional Chinese Medicine Agricultural Biogenomics, Changsha Medical University, Changsha, Hunan, China; 4 Key Laboratory of Swine Nutrition and Feed Science of Fujian Province, Key Laboratory of Swine Breeding in South China, Ministry of Agriculture and Rural Affairs, Aonong Group, Zhangzhou, China; 5 College of Agronomy, Hunan Agricultural University, Changsha, China

**Keywords:** alfalfa fermented feed, growth performance, meat quality, gut microbiota, Taoyuan Black pig

## Abstract

This study investigated the effects of substituting 50% basal diet with alfalfa fermented feed (AFF) on growth performance, meat quality, serum biochemical parameters, and gut microbiota in finishing-phase Taoyuan Black pigs. A total of 120 healthy pigs were randomly assigned to control (basal diet) and AFF groups (50% basal diet + 50% AFF), with 6 replicates per group (10 pigs/replicate). The experiment lasted 42 days. The results showed that, compared to the control group, the AFF group exhibited no significant differences in growth performance (*p* > 0.05) but had significantly lower serum triglycerides (TG), alanine aminotransferase (ALT), and malondialdehyde (MDA) levels (*p* < 0.05). In terms of meat quality, the substitution of AFF significantly increased muscle crude protein, energy levels, and the contents of glutamic acid, alanine, isoleucine, lysine, tyrosine, proline, total amino acids (TAA), umami amino acids (DAA), essential amino acids (EAA), and unsaturated fatty acids (UFA) (*p* < 0.05) while significantly decreasing the 45-min yellowness value (*p* < 0.05) and markedly reducing saturated fatty acid (SFA) content (*p* < 0.01). Regarding the gut microbiota, at the phylum level, the relative abundance of Bacteroidetes, Tenericutes, and Actinobacteria in the experimental group increased significantly (*p* < 0.05), Spirochaetes rose markedly (*p* < 0.01), and Proteobacteria decreased markedly (*p* < 0.01). At the genus level, the relative abundance of *Treponema*, *Ruminococcus*, and *Prevotella* increased significantly (*p* < 0.05). In conclusion, substituting 50% of the basal diet with AFF in late-stage Taoyuan Black pigs maintained growth performance while enhancing meat quality through improved muscle amino acid profiles and unsaturated fatty acid content. Additionally, it improved serum biochemical and antioxidant indicators and the cecal microbiota, promoting the overall health of the finishing pigs.

## Highlights

Replacing 50% of the basal diet with AFF can significantly improve the levels of muscle amino acids and fatty acids and the meat quality of Taoyuan Black pigs in the late fattening stage. This dietary change can also significantly enhance the serum biochemical and antioxidant indicators in fattening pigs and improve the cecal microflora, thereby promoting the overall health of the pigs.

## 1 Introduction

In recent years, the rapid development of animal husbandry in the majority of countries has resulted in a continuous increase in the demand for feed grains, making the supply–demand imbalance of feed grains one of the most prominent challenges to food security (1). To alleviate the gaps between raw material supply and demand, many countries have implemented initiatives to reduce and substitute corn and soybean meal in animal feed, urging the search for new feed grain reduction and substitution methods (2).

Alfalfa (*Medicago sativa* L.), renowned as the ‘King of Forage,’ exhibits strong stress resistance, rich nutritional content, high herbage yield, and excellent palatability (3, 4). Extensive research demonstrates that including alfalfa in the diets of various livestock species enhances growth performance and improves product quality (5–8). However, the predominant practice of adding alfalfa in the form of hay or meal to animal diets leads to nutrient loss, reduced palatability, and an impaired feed conversion rate during prolonged transportation/storage, ultimately affecting the production performance (9). Alfalfa fermented feed (AFF) maximally preserves nutritional components, conserves storage space, and facilitates transportation (10). Taoyuan Black pigs, a premium Chinese indigenous swine breed, possess several beneficial characteristics, including roughage tolerance, high fertility, strong adaptability, and tender meat quality. However, no studies have reported the use of fermented alfalfa in the production performance of Taoyuan Black pigs. In this trial, 50% of the basal diet for Taoyuan Black pigs in the late finishing stage was substituted with AFF to investigate its effects on growth performance, meat quality, gut microbiota, and related parameters, thereby providing a theoretical basis for the safe and efficient utilization of AFF in Taoyuan Black pigs.

## 2 Materials and methods

### 2.1 Ethical approval

All experiment procedures were reviewed and approved by the Animal Care Committee of the Institute, Changsha Medical University, Changsha, China.

### 2.2 Experimental design

This experiment employed a single-factor randomized trial design. A total of 120 healthy late-finishing Taoyuan Black pigs (all castrated male pigs) with similar body weights (76.56 ± 4.44 kg) were randomly assigned to two groups (6 replicates per group, 10 pigs per replicate). No significant differences existed in average initial body weight (IBW) between the groups (*p* > 0.05). The control group received a basal diet, while the experimental group received a 50% basal diet + 50% AFF.

The basal diet was formulated based on the nutritional requirements outlined in the NRC (2012) and the Chinese Local Pig Feeding Standards (NY/T65-2004), and the diet’s composition and nutrient levels are shown in Table 1. AFF was produced and supplied by Deren Animal Husbandry Technology Co., Ltd., using purple alfalfa as the raw material. The production process involved fine grinding of the alfalfa, mixing with dry supplements (straw, wheat bran, rice bran, soybean meal, corn, etc.), and anaerobic fermentation using multiple compound microbial strains (*Saccharomyces cerevisiae*, *Bacillus subtilis*, *Enterococcus lactis*), bioenzymes, polysaccharides, and other health factors. The AFF (fresh state) contained 56.92% moisture, 4.89% crude protein, 2.36% crude fat, 17.82% crude fiber, 6.29% crude ash, and 17.53 MJ/kg gross energy.

**Table 1:** Basal dietary nutrient levels (air-dry basis).

Items	Content (%)
Ingredients	
Corn	27.5
Rice bran	10
Rice bran meal	16
Second head	30
Soybean meal	12.5
Lys	0.55
Thr	0.05
Limestone	1.7
CaHPO_4_	0.4
Salt	0.3
Premix^1^	1
Total	100
Nutrient levels^2^	
DE (MJ/kg)	13.38
CP	15
CF	3.47
Ca	0.80
TP	0.76
AP	0.20
Lys	1.15
Met	0.25
Thr	0.60
Met+Cys	0.50

1 The premix provided the following per kg of diet: Vitamin A (VA) 12,000 IU, Vitamin D3 (VD3) 30,000 IU, Vitamin E (VE) 15 mg, Vitamin K3 (VK3) 3 mg, Vitamin B1 (VB1) 3 mg, Vitamin B2 (VB2) 6 mg, Vitamin B6 (VB6) 3 mg, Vitamin B12 (VB12) 24 μg, Copper (Cu) 15 mg, Zinc (Zn) 50 mg, Manganese (Mn) 10 mg, Selenium (Se) 0.3 mg, Iodine (I) 0.3 mg, Nicotinic acid 25 mg, and Pantothenic acid 20 mg.

2 The nutrient level is a calculated value.

Prior to the trial, pig pens, feed troughs, and water dispensers underwent thorough cleaning and disinfection using potassium permanganate fumigation. A 7-day pretest period preceded the 42-day formal trial. During the trial, each replicate group had ad libitum access to feed and water. The experimental group’s AFF (fresh state) was mixed with the basal diet at a 1:1 ratio through uniform stirring, ensuring consistent feeding management across the groups. Immunization protocols and pen disinfection strictly followed farm epidemic prevention regulations. Daily observations recorded feed intake, health status, pen temperature, humidity, and mortality/culling numbers.

### 2.3 Sampling and collection

At the beginning and end of the trial, fasted body weights of all 120 experimental pigs were measured to record initial and final body weights (IBW and FBW) and to calculate average daily feed intake (ADFI), average daily gain (ADG), and feed-to-gain ratio (F/G). Upon completion of the feeding trial, one pig per replicate was randomly selected for slaughter. Approximately 20 mL of blood was collected from the anterior vena cava of each pig and centrifuged at 3,000 rpm for 10 min at 4 °C to obtain serum, which was then aliquoted into centrifuge tubes and stored at −20 °C for subsequent analysis.

Following slaughter and dissection, cecal contents were immediately collected and divided into 5 mL centrifuge tubes, flash-frozen in liquid nitrogen, and subsequently transferred to a − 80 °C freezer for preservation. A 300 g sample of the longissimus dorsi muscle from the right-side carcass of each pig was collected at identical anatomical positions. Portions of the muscle samples were used onsite for meat quality determination, while the remaining portions were wrapped in aluminum foil and refrigerated for muscle nutrient composition analysis.

### 2.4 Serum index detection

Total protein (TP), albumin (ALB), glucose (GLU), triglycerides (TG), alanine aminotransferase (ALT), aspartate aminotransferase (AST), total cholesterol (TC), creatinine, and urea nitrogen were determined using a fully automated biochemistry analyzer (BS-600, Mindray Bio-Medical Electronics Co., Ltd., Shenzhen, China), with globulin (GLB) levels calculated accordingly. Total antioxidant capacity (T-AOC), superoxide dismutase (SOD), glutathione peroxidase (GPX), malondialdehyde (MDA), and glutathione (GSH) in pig serum were measured using commercial assay kits (Nanjing Jiancheng Bioengineering Institute, China) according to the manufacturer’s protocols, with a microplate reader (SpectraMax M5, Molecular Devices, USA).

### 2.5 Meat quality and nutritional composition

The PH (45 min and 24 h), meat color (45 min and 24 h), drip loss (45 min and 24 h), shear force (45 min and 24 h), and cooking loss (45 min and 24 h) of the longissimus dorsi muscle post-slaughter were measured according to NY/T821-2004 “Technical Specification for Determination of Pork Muscle Quality” and the relevant methodologies outlined in “Swine Production Science.”

The collected muscle samples were sectioned and stained, and myofiber characteristics were observed under a microscope using the following methods: Myofiber diameter: The longest distance between two points on the cross-section was measured as the major axis, while the shortest distance perpendicular to the major axis was defined as the minor axis. Multiple measurements were performed to calculate the average values. Myofiber density: The area of each field of view was calculated, and the number of muscle fibers within each field of view was counted.

The muscle samples were freeze-dried in a vacuum freeze dryer for 48 h and subsequently ground. Moisture, crude protein, crude fat, crude ash, and gross energy were determined, along with amino acid (fully automated amino acid analyzer, L-8900, Hitachi High-Technologies Corporation, Japan) and fatty acid contents (gas chromatograph, Shimadzu GC-14C, Shimadzu Corporation, Japan) in the longissimus dorsi muscle.

### 2.6 DNA extraction and PCR amplification

As previously reported, DNA extraction and 16S ribosomal RNA amplification were carried out. Fecal samples were processed for DNA extraction using the E.Z.N.A. ® Soil DNA Kit (Omega Biotek, Norcross, GA, USA) on the basis of the standard protocol. Using universal primers targeting the V3-V4 region 338F/806R, 16S rRNA from bacteria was amplified, and the samples were sequenced on an Illumina MiSeq PE300 platform (Illumina, SD, USA). Sequence reads from the original sequence were uploaded to the NCBI Sequence Read Archive.

### 2.7 Statistical analysis

After the preliminary processing of the experimental data using the Excel 2007 software, one-way ANOVA was performed with the SPSS 19.0 statistical software. If the difference between the groups was significant (*p* < 0.05), Duncan’s method was used for multiple comparisons. Values of 0.05 < *p* < 0.1 were considered indicative of a trend. The test results are presented in the form of mean ± standard deviation.

## 3 Results

### 3.1 Effects of AFF on the growth performance of the Taoyuan Black pigs during the late fattening period

As shown in Table 2, compared to the control group, the experimental group showed no significant differences (*p* > 0.05) in ADFI, F/G, IBW, FBW, or ADG.

**Table 2:** Effects of AFF on the growth performance of the finishing pigs (absolutely dry basis).

Items	Control group	Experimental group	*p*-value
Initial weight/kg	75.57 ± 2.65	77.56 ± 1.79	0.156
Final weight/kg	100.33 ± 3.45	102.10 ± 2.84	0.355
ADFI/kg	2.08 ± 0.031	2.06 ± 0.029	0.157
ADG/g	550.37 ± 40.02	545.19 ± 26.84	0.797
F/G	3.80 ± 0.24	3.78 ± 0.15	0.859

Different lowercase letters on the shoulder labels of the data in the same column indicate significant differences (*p* < 0.05), while the same letter or no letter indicates no significant difference (*p* > 0.05).

### 3.2 Effects of AFF on serum biochemical and oxidative/antioxidant indices in the Taoyuan Black pigs during the late fattening period

As shown in Tables 3, 4, substituting 50% of the basal diet with AFF significantly decreased serum TG and ALT levels by 27.85% (*p* < 0.05) and 27.86% (*p* < 0.05), respectively, compared to the control group. No significant differences were observed in TP, ALB, GLB, GSH, CHO, urea, Cre, or AST levels (*p* > 0.05). Regarding antioxidant status, the substitution markedly reduced MDA content by 66.24% (*p* < 0.05), while no significant effects were noted on GSH-Px, SOD, GSH, or T-AOC (*p* > 0.05).

**Table 3:** Effects of AFF on serum biochemical indices in the finishing pigs.

Item	Control group	Experimental group	*p*-value
TP (g/L)	90.86 ± 4.65	83.85 ± 11.22	0.292
ALB (g/L)	28.55 ± 0.66	27.39 ± 3.25	0.511
GLO (g/L)	62.31 ± 4.33	56.46 ± 8.02	0.247
GLU (mmol/L)	5.06 ± 0.43	4.81 ± 0.50	0.516
TG (mmol/L)	0.79 ± 0.089^a^	0.57 ± 0.05^b^	<0.01
CHO (mmol/L)	3.08 ± 0.61	3.27 ± 0.84	0.727
Urea (mmol/L)	6.71 ± 1.53	5.21 ± 0.49	0.110
Cre (μmol/L)	71.43 ± 9.12	63.27 ± 9.96	0.272
ALT (U/L)	69.03 ± 11.98^a^	49.80 ± 7.28^b^	0.034
AST (U/L)	54.20 ± 13.99	49.20 ± 9.00	0.570

Different lowercase letters on the shoulder labels of the data in the same column indicate significant differences (*p* < 0.05), while the same letter or no letter indicates no significant difference (*p* > 0.05).

**Table 4:** Effects of AFF on serum antioxidant indices in the finishing pigs.

Item	Control group	Experimental group	*p*-value
GSH-Px (U/mL)	749.03 ± 123.61	698.07 ± 87.58	0.526
SOD (U/mL)	281.26 ± 34.04	278.83 ± 11.93	0.904
CAT (U/mL)	0.16 ± 0.054	0.12 ± 0.02	0.209
GSH (μmol/L)	130.90 ± 26.29	141.78 ± 16.25	0.508
MDA (nmol/mL)	10.10 ± 3.54^a^	3.41 ± 1.64^b^	0.019
T-AOC (mmol/L)	0.30 ± 0.050	0.30 ± 0.03	0.916

Different lowercase letters on the shoulder labels of the data in the same column indicate significant differences (*p* < 0.05), while the same letter or no letter indicates no significant difference (*p* > 0.05).

### 3.3 Effects of AFF on the meat quality of the Taoyuan Black pigs during the late fattening period

As shown in Table 5, when AFF was used to replace 50% of the basal diet during the late fattening stage of the Taoyuan Black pigs, the backfat thickness of the pigs decreased by 10.15% and the carcass length increased by 6.76%, although these differences were not statistically significant (*p* > 0.05). Notably, the 45-min yellowness value decreased by 34.85% (*p* < 0.05), while the 45-min pH value exhibited an upward trend (*p* = 0.066). In addition, shear force, cooking loss, lightness, and redness at 45 min showed non-significant reductions (*p* > 0.05). No significant differences were observed in 24-h pH, lightness, redness, or yellowness between the treatment and control groups (*p* > 0.05).

**Table 5:** Effects of AFF on the quality of fattening pork.

Item	Control group	Experimental group	*p*-value
Backfat thickness/cm	3.94 ± 0.48	3.54 ± 0.28	0.108
Thick skin/cm	0.37 ± 0.11	0.39 ± 0.12	0.749
Length carcass/cm	90 ± 5.13	96.08 ± 7.72	0.139
Loin eye area/cm^2^	27.16 ± 5.01	27.03 ± 4.81	0.962
45-min Shear force/N	43.91 ± 8.39	38.68 ± 8.90	0.320
45-min cooking loss%	38.52 ± 3.81	35.52 ± 2.84	0.151
45-min pH	6.11 ± 0.19	6.33 ± 0.19	0.066
45-min L^*^_45min_	33.83 ± 4.29	31.57 ± 4.14	0.377
45-min a^*^_45min_	5.86 ± 1.61	5.13 ± 1.24	0.401
45-min b^*^_45min_	2.41 ± 0.47^a^	1.57 ± 0.46^b^	0.011
24-h Shear force/N	40.84 ± 12.96	36.55 ± 5.67	0.475
24-h cooking loss/%	36.74 ± 1.67	35.36 ± 1.11	0.122
24-h pH	5.95 ± 0.39	5.72 ± 0.094	0.206
24-h L^*^_24h_	35.75 ± 2.87	36.43 ± 3.35	0.714
24-h a^*^_24h_	6.25 ± 1.26	6.52 ± 1.48	0.739
24-h b^*^_24h_	2.26 ± 0.78	2.45 ± 0.67	0.653

Different lowercase letters on the shoulder labels of the data in the same column indicate significant differences (*p* < 0.05), while the same letter or no letter indicates no significant difference (*p* > 0.05).

### 3.4 Effects of AFF on the muscle fiber characteristics of the Taoyuan Black pigs during the late fattening period

**Figure 1:**
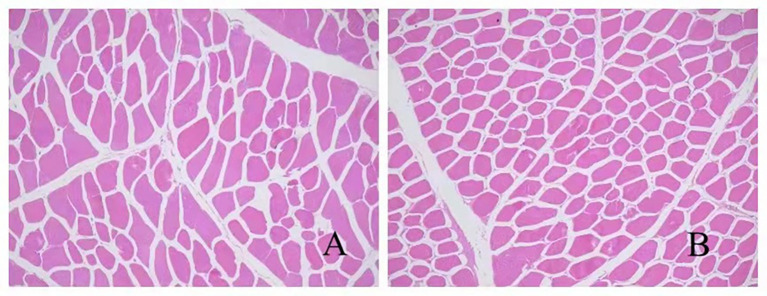
Muscle fiber section HE×100 **(A)** control group, **(B)** experimental group.

According to Table 6 and Figure 1, no significant differences were observed in muscle fiber diameter or density between the experimental group and the control group (*p* > 0.05), indicating that substituting 50% of the basal diet with AFF during the late fattening period of the Taoyuan Black pigs had no marked impact on muscle fiber characteristics.

**Table 6:** Effects of AFF on the muscle fiber characteristics of the finishing pigs.

Item	Control group	Experimental group	*p*-value
Myofiber density/μm	111.39 ± 17.18	105.47 ± 14.78	0.620
Myofiber diameter(/mm^2^)	88.60 ± 17.43	92.75 ± 15.41	0.734

### 3.5 Effects of AFF on the muscle nutrient composition of Taoyuan Black pigs during the late fattening period

According to Table 7, substituting 50% of the basal diet with fermented alfalfa during the late fattening period of the Taoyuan Black pigs significantly increased muscle crude protein and energy contents (*p* < 0.05), while crude ash content in muscles exhibited a decreasing trend (*p* = 0.055), although this difference remained statistically non-significant (*p* > 0.05).

**Table 7:** Effects of AFF on the muscle nutrient composition of the finishing pigs(%).

Item	Control group	Experimental group	*p*-value
CP	22.06 ± 0.99^b^	23.32 ± 0.68^a^	0.046
Ash	4.18 ± 0.11	3.49 ± 0.72	0.055
EE	5.07 ± 1.48	7.19 ± 2.97	0.147
DE KJ/g	16.01 ± 0.97^b^	18.69 ± 2.75^a^	0.049
Water Content	68.04 ± 3.99	65.41 ± 2.63	0.207

Different lowercase letters on the shoulder labels of the data in the same column indicate significant differences (*p* < 0.05), while the same letter or no letter indicates no significant difference (*p* > 0.05).

### 3.6 Effects of AFF on the muscle amino acid composition of Taoyuan Black pigs during the late fattening period

According to Table 8, substituting 50% of the basal diet with AFF during the late fattening period of the Taoyuan Black pigs significantly increased muscle glutamate, alanine, isoleucine, lysine, tyrosine, and proline contents by 6.08, 11.2, 13.64, 7.34, 15.91, and 17.33%, respectively (*p* < 0.05). Total amino acid (TAA), umami amino acid (DAA), and essential amino acid (EAA) contents in muscles were also markedly elevated (*p* < 0.05). Arginine content rose by 10.26%, showing a tendency to increase (0.05 < *p* < 0.1). No significant changes were observed in aspartate, glycine, tryptophan, methionine, valine, leucine, phenylalanine, threonine, histidine, cysteine, or serine contents (*p* > 0.05).

**Table 8:** Effects of AFF on amino acids in the muscle of the finishing pigs(mg/kg).

Item	Control group	Experimental group	*p*-value
Asp	2.09 ± 0.86	2.18 ± 0.16	0.378
Glu	3.62 ± 0.069^b^	3.84 ± 0.066^a^	<0.01
Gly	0.92 ± 0.064	0.96 ± 0.11	0.555
Ala	1.34 ± 0.083^b^	1.49 ± 0.010^a^	0.025
Try	0.20 ± 0.040	0.22 ± 0.040	0.372
Met	0.64 ± 0.032	0.69 ± 0.090	0.295
Val	1.11 ± 0.082	1.17 ± 0.071	0.276
Ile	1.10 ± 0.068^b^	1.25 ± 0.021^a^	0.042
Leu	1.86 ± 1.29	1.96 ± 0.088	0.235
Phe	1.17 ± 0.059	1.20 ± 0.054	0.412
Lys	2.18 ± 0.070^b^	2.34 ± 0.069^a^	0.019
Thr	1.09 ± 0.043	1.11 ± 0.022	0.339
His	1.07 ± 0.045	1.14 ± 0.076	0.13
Arg	1.56 ± 0.12	1.72 ± 0.083	0.067
Cys	0.17 ± 0.025	0.16 ± 0.047	0.537
Tyr	0.88 ± 0.047^b^	1.02 ± 0.070^a^	0.014
Ser	0.88 ± 0.057	0.91 ± 0.044	0.399
Pro	0.75 ± 0.049^b^	0.88 ± 0.026^a^	0.011
DAA	7.97 ± 0.20^b^	8.47 ± 0.20^a^	0.014
EAA	9.33 ± 0.34^b^	9.89 ± 0.27^a^	0.045
TAA	22.60 ± 0.49^b^	24.08 ± 0.78^a^	0.018

Delicious amino acids: Asp., Glu, Gly, and Ala; Essential amino acids: Try, Met, Val, Ile, Leu, Phe, Lys, Thr, and His.

Different lowercase letters on the shoulder labels of the data in the same column indicate significant differences (*p* < 0.05), while the same letter or no letter indicates no significant difference (*p* > 0.05).

### 3.7 Effects of AFF on the muscle fatty acid composition of Taoyuan Black pigs during the late fattening period

As shown in Table 9, substituting 50% of the basal diet with AFF during the late fattening period of the Taoyuan Black pigs significantly altered muscle fatty acid profiles. Saturated fatty acid (SFA) content decreased by 11.47% (*p* < 0.01), while unsaturated fatty acid (UFA) content increased by 4.57% (*p* < 0.05). Monounsaturated fatty acid (MUFA) content declined by 18.13% (*p* < 0.01), whereas polyunsaturated fatty acid (PUFA) content increased by 99.09% (*p* < 0.01). Notably, n-6 PUFA and n-3 PUFA contents increased by 94.15% (*p* < 0.01) and 195.02% (*p* < 0.01), respectively, leading to a 34.04% reduction in the n-6/n-3 PUFA ratio (*p* < 0.01).

**Table 9:** Effects of AFF on fatty acids in the muscle of the finishing pigs(%).

Item	Control group	Experimental group	*p*-value
Myristic acid (C14:0)	1.21 ± 0.029^a^	1.10 ± 0.060^b^	0.018
Palmitic acid (C16:0)	22.46 ± 0.69^a^	19.62 ± 1.12^b^	<0.01
Stearic acid (C18:0)	11.02 ± 0.69^a^	9.99 ± 0.076^b^	0.025
Eicosenoic acid (C20:1)	0.72 ± 0.052^a^	0.61 ± 0.044^b^	0.018
Palmitoleic acid (C16:1)	4.56 ± 0.18^a^	4.21 ± 0.095^b^	0.012
Oleic acid (C18:1n-9)	44.87 ± 1.55^a^	36.25 ± 1.68^b^	<0.01
Linoleic acid (C18:2n-6)	9.06 ± 0.27^b^	17.47 ± 1.04^a^	<0.01
α-Linolenic (C18:3n-3)	0.59 ± 0.029^b^	1.74 ± 0.076^a^	<0.01
γ-Linolenic (C18:3n-6)	1.70 ± 0.076^b^	3.64 ± 0.12^a^	<0.01
Arachidonic acid (C20:4n-6)	0.69 ± 0.067^b^	1.13 ± 0.083^a^	<0.01
SFA	34.69 ± 0.17^a^	30.71 ± 1.08^b^	<0.01
UFA	62.19 ± 1.47^b^	65.03 ± 0.91^a^	0.017
MUFA	50.15 ± 1.68^a^	41.06 ± 1.70^b^	<0.01
PUFA	12.04 ± 0.26^b^	23.97 ± 0.93^a^	<0.01
n-6PUFA	11.45 ± 0.25^b^	22.23 ± 0.93^a^	<0.01
n-3PUFA	0.59 ± 0.029^b^	1.74 ± 0.076^a^	<0.01
n-6PUFA / n-3PUFA	19.45 ± 1.05^a^	12.83 ± 0.78^b^	<0.01

SFA: Myristic acid, Palmitic acid, and Stearic acid; UFA: Eicosenoic acid, Palmitoleic acid, Oleic acid, Linoleic acid, α-Linolenic, γ-Linolenic, and Arachidonic acid; MUFA: Eicosenoic acid, Palmitoleic acid, and Oleic acid; PUFA: Linoleic acid, α-Linolenic, γ-Linolenic, and Arachidonic acid; n-3PUFA:α-Linolenic; n-6PUFA: Linoleic acid, γ-Linolenic, and Arachidonic acid.

Different lowercase letters on the shoulder labels of the data in the same column indicate significant differences (*p* < 0.05), while the same letter or no letter indicates no significant difference (*p* > 0.05).

Myristic acid, stearic acid, cis-11-eicosenoic acid, palmitoleic acid, and oleic acid contents were significantly decreased (*p* < 0.05), while palmitic acid content was markedly reduced (*p* < 0.01). Conversely, linoleic acid, *α*-linolenic acid, *γ*-linolenic acid, and arachidonic acid contents were dramatically elevated (*p* < 0.01).

### 3.8 Effects of AFF on the gut microbiome of Taoyuan Black pigs during the late fattening period

**Figure 2:**
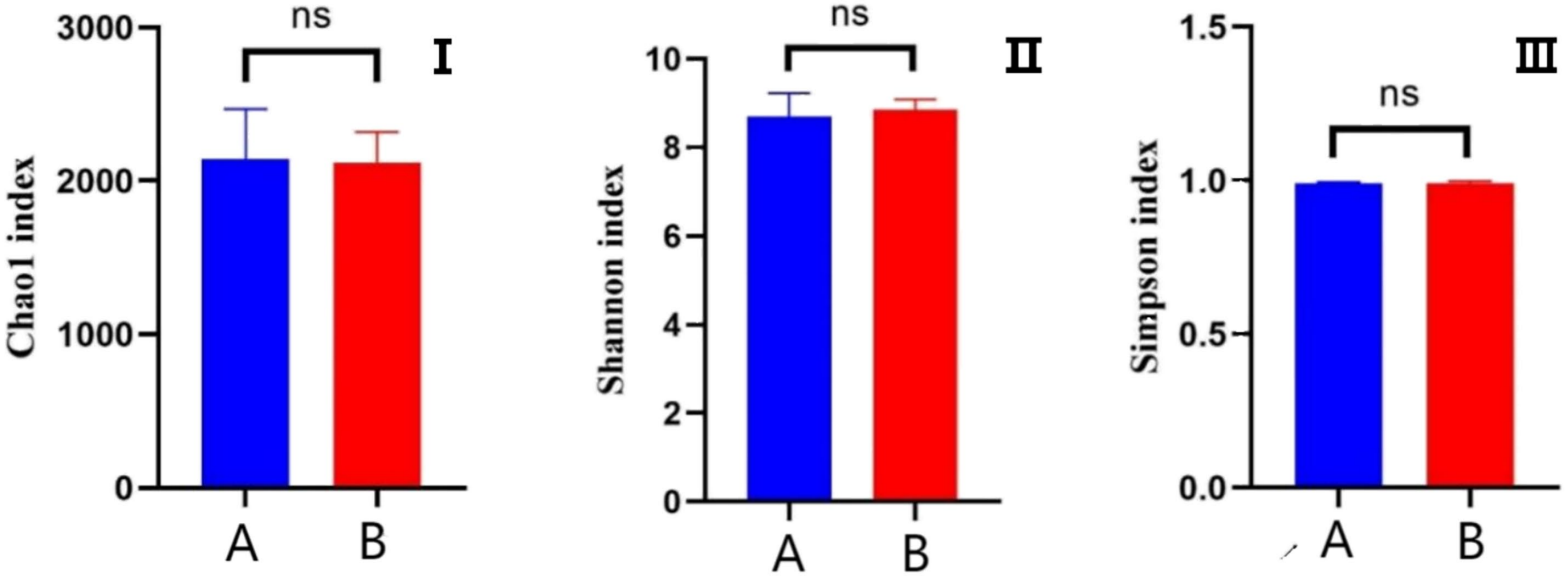
Analysis of *α*-diversity of the cecal microbiota (**I**: Chao 1 index; **II**: Shannon index; **III**: Simpson index; A: control group, B: experimental group).

**Figure 3:**
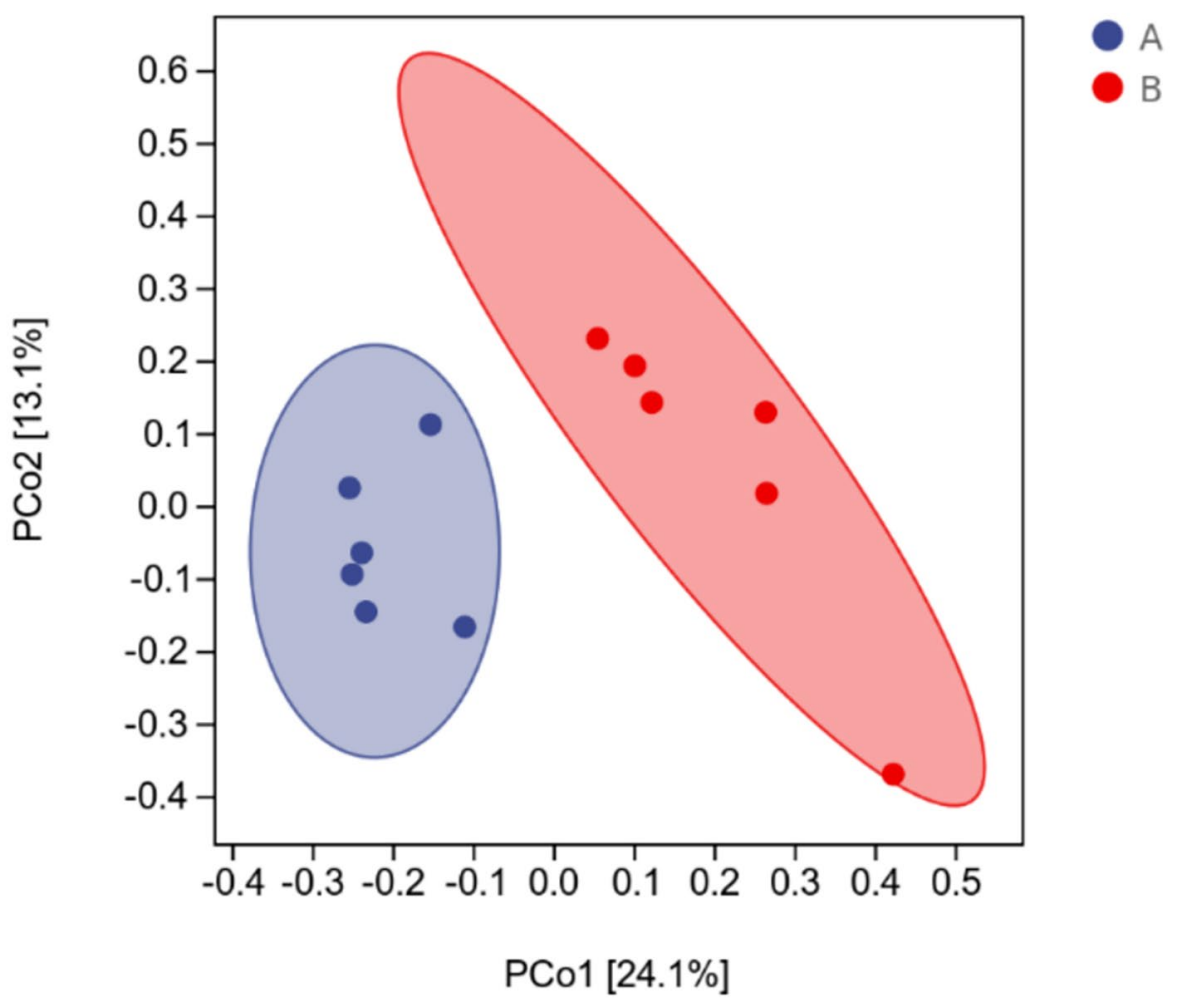
Cecal microbial PCoA analysis (A: control group, B: experimental group).

**Figure 4:**
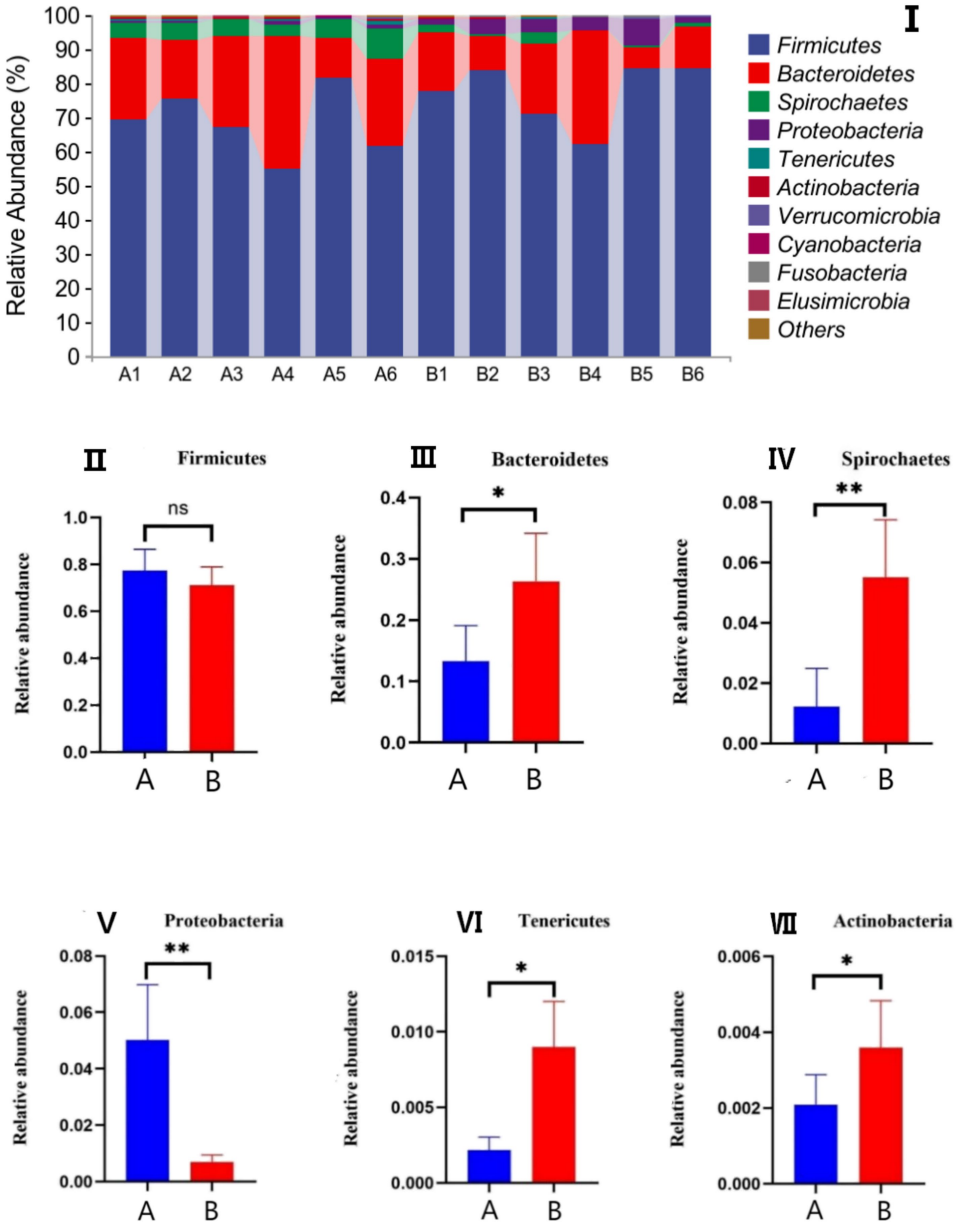
Effects of AFF on the relative abundance of the gut microbiota at the phylum level **(I)** and its dominant bacteria **(II–VII)**. (A: control group, B: experimental group).

**Figure 5:**
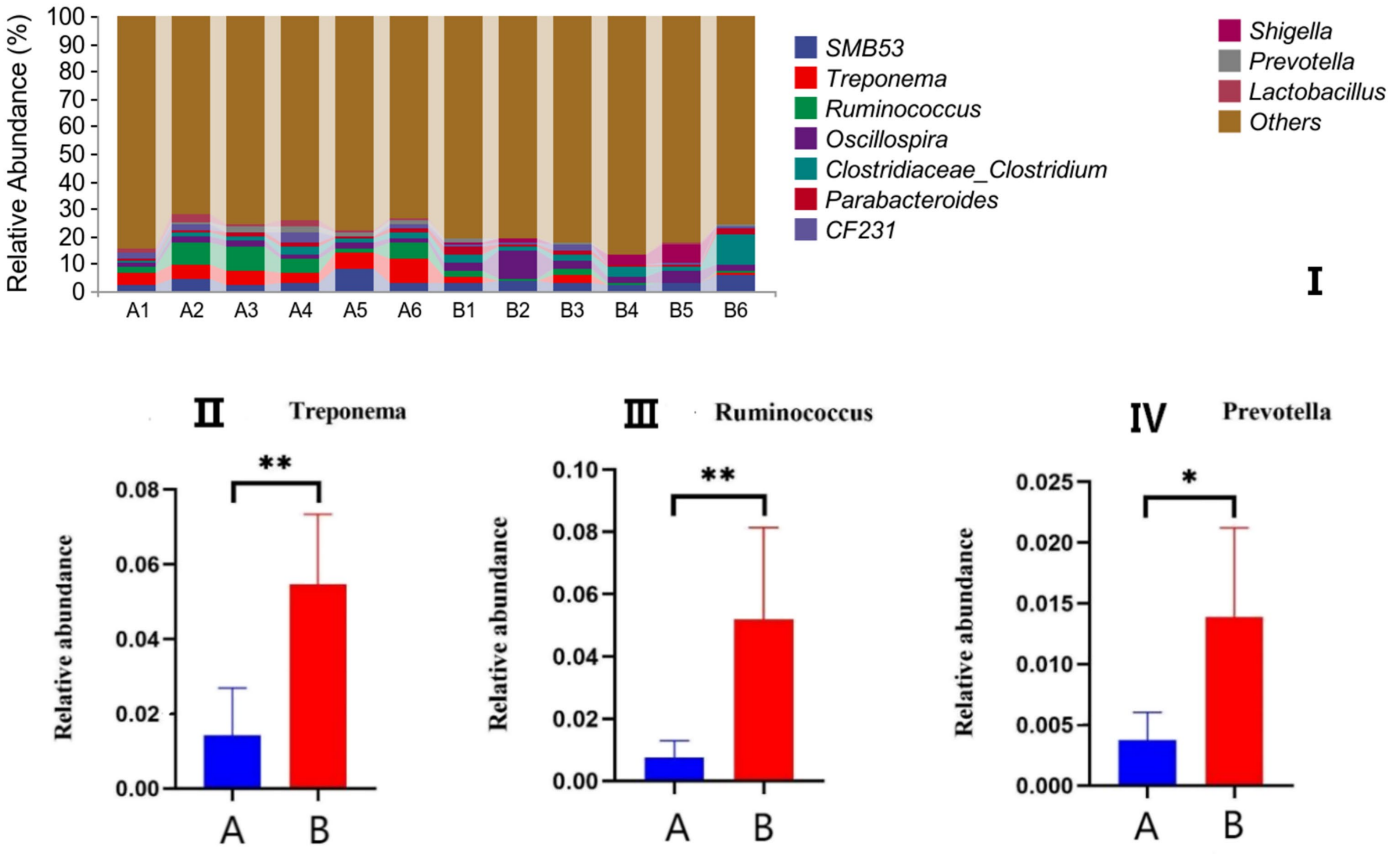
Effects of AFF on the relative abundance of the gut microbiota at the genus level **(I)** and its dominant bacteria **(II–VII)**. (A: control group, B: experimental group).

The effects of AFF on the intestinal microbiota of the Taoyuan Black pigs during the late fattening period are presented below. As shown in Figure 2, AFF had no significant impact on the Chao1, Simpson, or Shannon indices (*p* > 0.05). Furthermore, distinct differences in the community structure were observed between the control and AFF groups (Figure 3). As illustrated in Figures 4, 5, the dominant bacterial phyla in the cecum of the Taoyuan Black pigs during the late fattening period were Firmicutes and Bacteroidetes. At the phylum level, compared to the control group, no significant change was observed in Firmicutes (*p* > 0.05), while the relative abundance of Bacteroidetes, Tenericutes, and Actinobacteria increased significantly (*p* < 0.05). The relative abundance of Spirochaetes exhibited a pronounced increase (*p* < 0.01), whereas Proteobacteria decreased markedly (*p* < 0.01). At the genus level, the relative abundance of *Treponema*, *Ruminococcus*, and *Prevotella* was significantly elevated (*p* < 0.05).

## 4 Discussion

Optimizing the feed conversion ratio and enhancing the growth performance serve as pivotal strategies for reducing production costs and improving economic efficiency in livestock operations (11). However, the direct inclusion of alfalfa in monogastric animal diets may reduce digestibility due to its high crude fiber content, which can subsequently compromise growth performance (12). Gao et al. (13) reported that substituting 50% of the diet with alfalfa decreased carcass weight in Tibetan pigs. Similarly, Wüstholz et al. (14) demonstrated that while ensiled alfalfa could serve as a protein supplement for finishing pigs, excessive dietary fiber negatively impacted growth performance. In contrast, microbial fermentation partially degrades crude fiber and reduces anti-nutritional factors (15), thereby enhancing digestibility and producing fermented feed-specific aromatic compounds that improve palatability (16).

Therefore, AFF may be more suitable for monogastric animal diets. Luo et al. (17) showed that replacing 8% of corn and soybean concentrate protein with fermented alfalfa meal significantly increased ADFI in weaned piglets, although FBW, ADG, and F/G remained unaffected. In this study, substituting 50% of the basal diet with AFF in late-fattening Taoyuan Black pigs had no adverse effects on ADFI, F/G, IBW, FBW, or ADG. Although growth performance was not improved, the lower cost of AFF compared to the basal diet suggests substantial practical application potential (18). Nevertheless, the optimal inclusion level for maximizing economic benefits warrants further investigation.

Serum biochemical parameters and antioxidant levels serve as crucial integrated indicators reflecting an animal’s physiological status, health condition, and nutrient digestion/metabolism (19). Among these, TG and TC (20) represent key biomarkers of lipid metabolism, with lower concentrations typically indicative of enhanced fat utilization efficiency. ALT and AST (21) are utilized to assess protein digestion and metabolism while also serving as important indicators of hepatocellular damage and stress response. MDA (22), a lipid peroxidation product generated during free radical-mediated degradation of plasma lipids, directly reflects the extent of cell membrane oxidative damage and is widely employed for the quantitative evaluation of OS. Consistent with previous findings, Li et al. (23) demonstrated that AFF reduced MDA content in Muscovy duck muscle, along with serum TG and AST levels. He′s research (24) confirmed that dietary incorporation of 50 g/kg alfalfa meal decreased serum TG concentration. Similarly, Dabbou et al. (25) reported that dietary supplementation with alfalfa flavonoids reduced MDA levels in rabbit meat. The present study aligns with these established observations. Partial replacement of concentrate with 50% AFF in finishing pig diets significantly reduced serum TG, ALT, and MDA concentrations. This suggests that AFF may enhance physiological functions through improved lipid metabolism, hepatoprotective effects, and augmented antioxidant capacity, thereby exerting beneficial impacts on swine health and production performance.

AFF significantly enhances pork quality in finishing pigs through its rich bioactive compounds (e.g., saponins, polysaccharides, and flavonoids). These compounds exert protective effects by scavenging free radicals and inhibiting lipid peroxidation, thereby preserving the structural integrity of the myocyte membrane and reducing protein denaturation (26). This mechanism maintains the physical properties of muscle fibers. In this study, dietary supplementation with AFF showed no significant effect (*p* > 0.05) on the shear force of the longissimus dorsi muscle, indicating limited impact on meat tenderness. However, it significantly increased the yellowness of meat color at 45 min post-slaughter (*p* < 0.05), suggesting improved myoglobin stability or pigment deposition. These findings align with Wang et al.’s (27) research on sweet sorghum-alfalfa co-silage in meat lambs, confirming its positive regulatory role in meat color attributes.

In terms of nutritional composition, the content of crude protein, eight EAAs, and various fatty acids in muscle directly correlates with its nutritional value and flavor characteristics. In this trial, AFF significantly increased the crude protein content and energy level in the longissimus dorsi muscle of the finishing pigs, aligning with the findings of Li et al. (28), where fermented sorghum distillers’ grains enhanced crude protein in finishing pigs. The underlying mechanism may be attributed to microbial degradation of crude fiber and increased microbial protein synthesis during fermentation, thereby optimizing protein utilization efficiency. Furthermore, dietary supplementation with AFF significantly elevated the content of DAAs, EAAs, and TAAs in the longissimus dorsi muscle. Specifically, glutamate and alanine (key umami-enhancing amino acids), isoleucine and lysine (essential amino acids), tyrosine, and proline exhibited significant increases. These results are consistent with Ding’s research (29), indicating that alfalfa and its fermented products improve the muscle amino acid profile through their balanced amino acid composition and the bio-transformation of nutrients during fermentation. Concurrently, AFF enhanced the content of PUFAs, such as linoleic acid and arachidonic acid, while significantly reducing SFAs, including myristic acid, palmitic acid, and stearic acid. This synergistic modulation further enhances pork quality. In summary, incorporating AFF into finishing pig diets enhances both the nutritional value and flavor attributes of the longissimus dorsi muscle.

The porcine gastrointestinal tract harbors a complex microbial ecosystem comprising bacteria, fungi, protozoa, viruses, and bacteriophages, which collectively play a pivotal role in host health and productivity. A balanced gut microbiota signifies an optimal physiological state. Previous studies (30) demonstrated that dietary inclusion of 30% alfalfa improved meat quality in Heigai pigs by modulating gut microbiota composition without compromising growth performance. Similarly, Xu et al. (31) reported that alfalfa silage enhanced meat quality by remodeling the gut microbiota and altering SCFA profiles. In the present study, the relative abundance of Firmicutes remained unaffected, whereas supplementation with AFF significantly increased the relative abundance of Bacteroidetes—a shift associated with reduced adipogenesis in finishing pigs (32). In addition, the relative abundance of Spirochaetes, Tenericutes, and Actinobacteria was markedly elevated. Taxonomic analysis at the genus level revealed significant increases in *Treponema*, *Ruminococcus*, and *Prevotella*. These findings collectively indicate that AFF enhances the species richness of the cecal microbiota in finishing pigs, potentially attributable to the functional components within the AFF matrix.

## 5 Conclusion

Collectively, dietary supplementation of the basal diet with 50% AFF significantly reduced muscle b* values and the concentrations of SFA and MUFA while elevating crude protein and energy densities and augmenting the levels of several amino acids and PUFAs in the longissimus dorsi muscle of the Taoyuan Black pigs, without compromising growth performance, thereby conferring a moderate improvement in meat quality. In addition, AFF markedly decreased serum triglycerides, alanine aminotransferase, and malondialdehyde concentrations; ameliorated cecal microbial dysbiosis; and consequently enhanced the metabolic health of the finishing pigs.

## Data Availability

The data presented in this study have been deposited in the Sequence Read Archive (SRA) under BioProject accession PRJNA1359608.
